# Secretome Analysis: Reading Cellular Sign Language to Understand Intercellular Communication

**DOI:** 10.1016/j.mcpro.2023.100692

**Published:** 2023-12-10

**Authors:** Wei Wu, Jeroen Krijgsveld

**Affiliations:** 1 Singapore Immunology Network (SIgN), Agency for Science, Technology and Research (A∗STAR), Singapore, Singapore; 2 Department of Pharmacy, National University of Singapore, Singapore, Singapore; 3 Division of Proteomics of Stem Cells and Cancer, German Cancer Research Center (DKFZ), Heidelberg, Germany; 4 Medical Faculty, Heidelberg University, Heidelberg, Germany

**Keywords:** cellular secretion, click-chemistry, *in vivo* secretome labeling, affinity mass spectrometry, glycosylation, inter-cellular communication

## Abstract

A significant portion of mammalian proteomes is secreted to the extracellular space to fulfill crucial roles in cell-to-cell communication. To best recapitulate the intricate and multi-faceted crosstalk between cells in a live organism, there is an ever-increasing need for methods to study protein secretion in model systems that include multiple cell types. In addition, posttranslational modifications further expand the complexity and versatility of cellular communication. This review aims to summarize recent strategies and model systems that employ cellular coculture, chemical biology tools, protein enrichment, and proteomic methods to characterize the composition and function of cellular secretomes. This is all geared towards gaining better understanding of organismal biology *in vivo* mediated by secretory signaling.

## Highlights

• Cell type-specific and proximity biotin labeling enables secretome characterization.
• Glycan-mediated secretome analysis requires carefully controlled interpretation.
• Recent trends to perform secretome analysis *in vivo*.
• Computational methods are critical to interpret cellular and organismal secretion.

## Graphical Abstract

**Figure:**
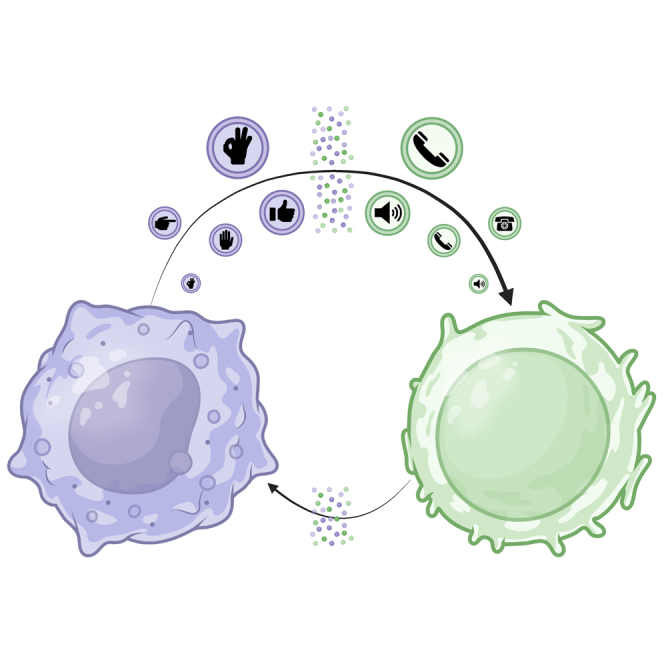

## In Brief

Probing intercellular crosstalk mediated by secreted proteins requires specific mass spectrometry strategies, metabolic labels, and molecular tools. This review aims to summarize analytical strategies that have emerged recently for the enrichment and characterization of secreted proteins, including their posttranslational modifications. We discuss the application of such strategies towards the recapitulation of systemic cellular crosstalk *in vivo*.

## The Secreted Proteome

The term secretome was first used in the year of 2000 to designate the secretory processes in the bacteria *Bacillus subtilis* (1) and has since become a generally used term to describe the global group of proteins that are secreted, released, or shed into the extracellular environment by a cell, tissue, organ, or organism at any given time. It has been estimated that the mammalian proteome encompasses nearly 3000 secreted proteins and >2000 that are localized to the plasma membrane, collectively representing some 25% of the proteome (2, 3). In addition, trafficking these proteins through the secretory pathway is an energetically expensive process, estimated to involve >250 proteins that are needed after the completion of protein synthesis to translocate secretory proteins through the endoplasmic reticulum (ER) and Golgi, while assembling disulfide bonds and adding N- and O-linked glycans (4). This expenditure is warranted by the fact that secretory proteins fulfill a range of important extracellular functions that are key to proper functioning of the cell in its niche, organ, and eventually in the entire organism.

Specifically, the secretome comprises a large variety of bioactive molecules such as enzymes, hormones, antibodies, and extracellular matrix (ECM) proteins that provide a scaffolding function in the ECM and that play important roles in regulating cell–cell and cell–ECM interactions (3, 5, 6). In addition, an important class of proteins is constituted by growth factors and cytokines that signal to recipient cells to induce a specific response. This is not only required to maintain tissue homeostasis in healthy tissue (as further stipulated below) but is also crucial in diseases like cancer where secretory proteins constitute the organizing principle among cancer cells, stroma, and immune cells in the tumor microenvironment (7, 8, 9). Knowing and understanding the proteins in the extracellular space that sustain the mutual interactions among cells therefore not only provides insight in fundamental aspects of tissue maintenance and onset of disease but may also provide clues for intervention by interfering in this network (7, 10). Furthermore, it is worth noting that proteins in body fluids like blood plasma and cerebrospinal fluid are all secreted by cells and organs throughout the body, putting secretory proteins at center stage for biomarker discovery. The aim of this review is to provide an overview of proteomic strategies that have emerged recently for the enrichment and characterization of secreted proteins, including their posttranslational modifications. Furthermore, we discuss the application of such strategies towards the recapitulation of systemic cellular crosstalk *in vivo*.

## Protein Secretion in Health and Disease

**Fig. 1:**
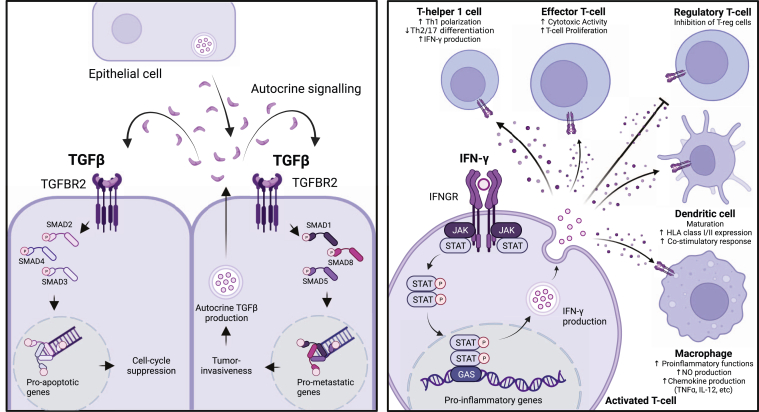
**Autocrine and paracrine signaling mediated for secretion.** (*Left*) Autocrine effects of TGFβ at tumor initiation and during metastasis. (*Right*) Paracrine effects of IFN-γ on immune cell populations.

Secretory proteins can either function in an autocrine or paracrine fashion to sustain a host of processes that are essential for tissue homeostasis, development, and response to stress. In autocrine signaling, a cell produces a soluble mediator into the extracellular space that binds to a receptor on the same cell, thereby completing a self-sufficient form of auto-regulation. For instance, autocrine production of transforming growth factor-beta (TGFβ) in cancer is a well-studied yet intriguing case of bi-phasic self-regulation (Fig. 1, Left). In the early stages of cancer, TGFβ is tumor-inhibitory by suppressing cell cycle progression and promoting apoptosis. However, in the late stages, TGFβ turns to increase tumor invasiveness and metastasis (11). Paracrine signaling is mediated by soluble factors produced by one cell and acting on another within the diffusion radius to enable signaling outcome that is dependent on another cell population and allows for a more complex and fine-regulated signaling network with more regulatory points. For instance, interferon-gamma (IFNγ) is one of the most well-understood paracrine mediators in the human body (Fig. 1, Right). While specialized production of IFNγ is restricted to immune cells, almost all other cell types in the body have IFNγ receptors to receive and react to IFNγ (12). This ensures that immune cells can effectively modify the tumor microenvironment for successful elimination of threats in the body.

An important and extensively studied function of cellular secretion is tissue repair. Since a large repertoire of secreted proteins has documented functions that promote proliferation and migration, it is congruent that cellular secretions can stimulate wound healing in skin regeneration (13). On the other hand, preconditioning of mesenchymal stromal cells was shown to produce different secretomes, that may be of therapeutic use (14), alluding to the possibility of using cellular secretomes produced under certain stimuli to treat diseases. Particularly in body systems where repair is desirable yet naturally inefficient, the utility of secreted factors have been heavily investigated. For instance, mesenchymal stem cell secretomes have been explored in therapies for trauma, stroke, or neurodegenerative conditions such as spinal cord or traumatic brain injuries, ischemic stroke, and Parkinson’s disease (15). Skeletal muscles have also been shown to secrete different sets of proteins depending on the state of physical exercise, during myogenesis, in dystrophin deficiency, muscle atrophy, or insulin stimulation (16). Moreover, stem cell secretomes have also been tested in tendon and ligament treatment (17). Collectively, these examples demonstrate that cellular secretions may be a rich source of potential therapeutics for use in regenerative therapy.

Not only do secretomes support tissue healing, secreted proteins can also regulate tissue organization and remodeling. For instance, autocrine signaling is important in myocardial biology and diseases, and secreted factors such as FGF2, VEGF, and IL-11 are critically required for cardiac remodeling (18). Dysregulation of such autocrine signaling can manifest in pathophysiological mechanisms such as hypertrophy, fibrosis, and inflammation. Paracrine signaling mediated by secreted proteins are also critical in mammary gland development and tissue organization, which are dysregulated in breast cancer (19). The pancreas is an important endocrine organ, in which alpha-cells and beta-cells are thought to take on mutually exclusive roles in glucagon and insulin production. Yet, in such histological proximity, alpha cells have been shown to engage in paracrine signaling, to indirectly regulate beta-cell production of insulin (20). Such examples of developmental crosstalk mediated by secretions are very prevalent in animals. In fact, for a long time, autocrine signaling was thought to be limited to large animals, whereas bacteria species exclusively rely on quorum sensing to communicate. Recent studies have unified these two mechanisms and shown that similar types of genetic circuits control many autocrine and quorum-sensing cells and that these are essentially two extreme ends of a common spectrum of crosstalk (21).

Secreted proteins are not only crucial in tissue homeostasis, but they are also key players in pathological processes. This notably includes cancer, where the tumor microenvironment constitutes a niche of cancer cells, stroma, and immune cells that closely interact by releasing various cytokines, chemokines, and other factors to play a decisive role in the survival and progression of tumors (22, 23). Additionally, tumors exploit ECM remodeling by the release of various proteases and protein-modifying enzymes that affect ECM stiffness, which in turn can drive malignant transformation and tumor growth, enhance epithelial-to-mesenchymal transition and metastasis, and promote drug resistance (24). Immune cells are essential components of the tumor microenvironment, where mounting evidence indicates that both innate and adaptive immune cells play a role in promoting tumor progression (25). Collectively, gaining insights into these interactions mediated by cell-secreted factors will pave the way for improved therapeutic approaches that target multiple components of the tumor microenvironment simultaneously, increasing the likelihood of the design of effective therapeutic interventions for patients (26, 27, 28).

As if secretion-mediated crosstalk is not complex enough, proteins in the extracellular space may also be functionally different, as dictated by different extracellular protein interactions, and different substrates present uniquely in the extracellular space. This moonlighting adds yet another layer of mystery to understand the mechanisms of intercellular crosstalk mediated by cellular secretions (29).

## Mechanisms of Protein Secretion

Protein secretion to the cell exterior can be generally distinguished to proceed by either classical or non-classical ways. Classically secreted proteins are directed to the cell exterior by signal peptides (30). These are typically short sequences of 16 to 30 amino acids at the protein N terminus that shuttle the newly synthesized polypeptide chains towards the secretory path. The signal peptide usually consists of a positively charged n-region, a hydrophobic h-region, and a signal peptidase recognition site (31, 32), at which the protein destined for secretion will be cleaved after plasma membrane fusion with the cell exterior. The signal peptide steers translocation of the nascent proteins into the ER and subsequently to the Golgi apparatus, where critical posttranslational modifications including cleavage and functionalization may occur. During transit, glycosylations are added, processed, and trimmed in the Golgi apparatus, to endow function and boost protein stability in the harsh extracellular environment (33). As fully modified and glycosylated proteins bud off the trans-Golgi network as secretory vesicles, COPII and Rab proteins then direct the fusion of secretory vesicle membranes with the plasma membrane, through which the proteinaceous contents of secretory vesicles are then released to the cell exterior (34).

Although the majority of proteins follow this classical pathway, as much as 30 to 40% of documented secreted proteins do not contain identifiable signal peptides and are secreted by various non-classical mechanisms that bypass the Golgi (35, 36, 37). Non-classical secretion modes such as in the case of ‘Golgi bypass’ are increasingly reported and emerging as not so unconventional as previously anticipated (38). In addition, other types of protein secretion have been documented through receptor ectodomain shedding, a process where the extracellular domain of a protein with plasma membrane fate may be cleaved by membrane-resident enzymes or other proteolytic enzymes secreted into the extracellular space (39). A mechanistically well-studied example of this is the γ-secretase cleavage and shedding of amyloid precursor protein ectodomains that results in the pathological accumulation of amyloid peptide plagues in Alzheimer’s disease (40).

**Fig. 2:**
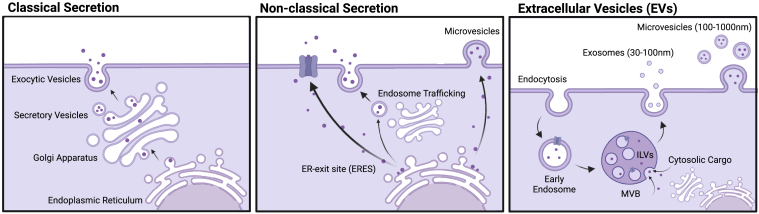
**Modes of secretion.** (*Left*) Classical secretion *via* Golgi apparatus. (*Middle*) Nonclassical nodes of secretion that bypass the Golgi apparatus. (*Right*) Extracellular vesicle secretion through formation of multivesicular bodies (MVB) and intralumenal vesicles (ILVs).

In the last decade, secretion of extracellular vesicles (EVs) garnered a lot of attention, as these may become excellent biomarkers in liquid biopsies and explain organotropic distribution of cancer metastasis (41). EVs are membrane-bound at the point of release and clearly distinct in the mechanism of secretion, compared to classical or Golgi-independent secretion, where free proteins are released instead into the extracellular space (42) (Fig. 2). It is key to note that due to the biogenesis path of EVs, these can retain a relatively controlled environment for the encapsulated proteins and may still protect the signaling state of secreted molecules even outside the source cells (43). EV secretion is not templated in sequence and also highly dependent on the cell state, fitness, and external stimuli and as such poorly predictable. Thus, changes in EV secretion can be extremely dynamic in response to different cellular states and molecular triggers.

In all three modes of secretion discussed above, it is important to consider the secretion flux and whether the secretion is constitutive or inducible. Most cells have a basal level of constitutive secretion. The biological function of such secretion is likely homeostasis or autocrine stimulation within the tissue environment. Inducible secretion usually involves triggered release of presynthesized extracellular protein loads and can be observed in neuronal signal transduction such as neurotransmitters (44) or in entero- and neuro-endocrine regulation (45, 46). Neuroendocrine secretion broadly controls human behavior through coordinating motor movements, language and affective behavior, but is also a key trigger of the immune system to respond to stimuli that necessitate an immunological response. The neuroendocrine and enteroendocrine systems together also control diet and metabolism, for systemic regulation of energy balance and homeostasis.

## Analytical Strategies for Secretome Characterization

**Fig. 3:**
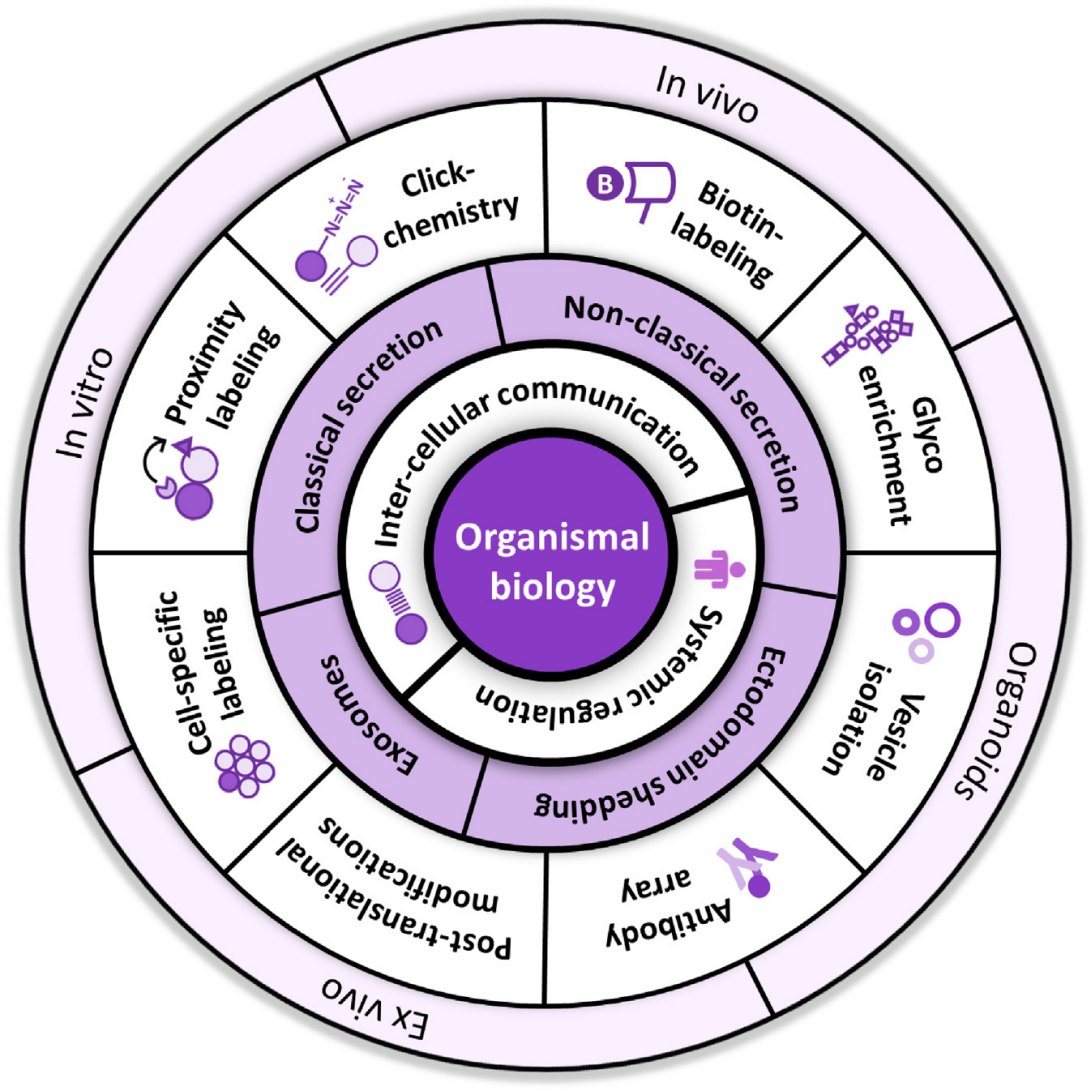
**Analytical strategies in secretome characterization**.

The evident importance of secreted proteins in many aspects of organismal biology has prompted the development of a range of methods to determine the composition of cellular secretomes (Fig. 3). Since this typically cannot be done directly *in vivo* because of the complexity of organ systems involving multiple cell types, cell culture *in vitro* has been a useful model system for secretome analysis either in mono-culture of specific cell types or, increasingly, in coculture systems. In addition, a variety of approaches have been introduced to enrich secretory proteins to enhance analytical depth, either by exploiting protein modifications as an affinity handle or by designing sophisticated tools in chemical biology for protein labeling. Finally, when combined with genetic engineering, some of these methods are starting to be used to characterize secretomes *in vivo*. We here describe the most salient methods introduced in the recent literature (Fig. 3), highlighting their methodological concepts, merits, and application to biological questions, and indicating challenges for the future.

## Secretome Analysis by Serum Starvation

*In vitro* cell culture has been a long-standing model system to identify the repertoire of proteins released by specific cell types, despite the general shortcoming of 2D cell culture as a simplified representation of the cellular environment *in vivo*. A major bottleneck in the characterization of secretory proteins in cell culture experiments is the presence of bovine serum constituents in culture media. For a quantitative perspective, *in vitro* secretomes can reach a concentration of 0.05 to 0.1 μg/μl without stimulation, divided over hundreds to thousands of different protein species. Full culture medium with 10% bovine serum can reach concentrations of 5-6 μg/μl, where >95% of the proteins are the top 10 abundant blood proteins. This drastic imbalance in protein abundance creates the problem that low abundance–secreted proteins may be masked by high abundance artefacts, thereby hampering their detection and identification by mass spectrometry. This may be partially circumvented by omitting serum; however such serum starvation may create a stress condition that distorts secretome composition (47, 48). Use of SILAC-labeled cells is an effective way to distinguish residual serum from cellular proteins. This has been employed, for example, to investigate substrates of beta-secretase 2 (BACE2) in a model for cancer metastasis in melanoma (49) or to show that the secretome pattern of (SILAC-labeled) astrocytes was altered within a few hours, depending on the type of (unlabeled) neurons that were added in coculture, including proteins that were secreted by classical, unconventional, and shedding mechanisms (50). To partially circumvent potential negative effects due to extended periods of serum starvation, one approach is to decouple cell treatment (exposure to cytokines) from serum depletion, by only removing serum from growth media during the last 2 h before harvest of conditioned media (51). In this way, the authors could identify profiles of cytokine-induced protein secretion over a 72 h period.

Even after eliminating bovine serum, trace amounts of remnant bovine proteins may still be identified by increasingly sensitive LC-MS/MS. In these cases, the consensus in the field is to search the data against a database of the experimental model system (*i.e.*, human, mouse, etc) concatenated with bovine serum protein sequences, such that any hit to the bovine proteins can be removed. The caveat in this procedure is that proteins in the experimental model system that share significant bovine similarity are also eliminated, calling for some caution.

## Enrichment of Secreted Proteins with Clickable Amino Acids

Compared to eliminating bovine serum altogether, more powerful alternative approaches have been devised to selectively enrich for secreted proteins originating from cells cultured in the presence of serum. Such enrichment strategies typically display significantly enhanced secretome coverage and analytical depth. As a general concept, these strategies exploit the intrinsic cellular biosynthetic machinery to incorporate non-natural amino acids into nascent proteins *via* clickable reactive groups (*i.e.* azides or alkynes), using the bioorthogonal non-canonical amino acid tagging (BONCAT) approach (52). Methionine labeling with azido-homoalanine (AHA) has been immensely useful to specifically capture low quantities of secreted proteins from conditioned media (48, 53). Alkyne-containing homo-propargylglycine is another alternative non-natural amino acid that is compatible with BONCAT. Minimal labeling with AHA can ensure the incorporation of a first AHA at the initiator codon, and on average 2 to 3 other methionines, to still retain good accessibility of the protein for on-bead trypsin digestion, followed by LC-MS/MS for protein identification.

An attractive feature of click chemistry is that chemical coupling of an azide-bearing protein to alkyne beads is highly selective and creates a covalent bond that allows stringent washing to remove non-labeled background proteins, such as serum contaminants. Alternatively, coupling can occur to alkyne-biotin for subsequent enrichment of the azide-modified protein pool using avidin affinity purification. Pulse labeling of cells with AHA has been applied to characterize the secretome composition of macrophages, and this was combined with SILAC labeling to quantify changes in protein secretion upon macrophage activation (48). Moreover, the duration and time window of protein labeling can be chosen as desired, to identify proteins that are secreted immediately (within hours) as a primary response to the cellular perturbation or rather in a delayed manner to investigate adaptive or compensatory processes (48). This approach has been used in various biological contexts, for example, to characterize stage-specific secretome profiles of mammalian cells during bacterial infection (54). In addition, it was applied to identify secretory proteins with functional relevance in disease, such as the hypoxia-induced secretion of PCSK6, which was then shown to serve as a marker for myocardial infarction to mediate cardiac remodeling (55). Furthermore, complement factor B was shown to be prevalent in the microenvironment of pancreatic ductal adenocarcinoma and was associated with poor patient survival, potentially explained by complement factor B’s function in promoting cell proliferation (56). Beyond cell lines, AHA labeling has been applied to organoids, showing secretion of several dozen proteins in mouse prostate organoids that depends on the presence of ETS-related gene, a frequently re-arranged gene in prostate cancer, identifying various factors mediating cell signaling and adhesion with potential autocrine or paracrine roles in tumor onset (57).

## Secretome Analysis in Co-culture Systems

### Label-Free Approaches

A core function of secreted proteins is to mediate communication between cells in a multicellular and potentially heterogeneous environment. The simplest experimental set-up to identify factors that confer such cross-talk uses transfer of conditioned medium obtained from one cell culture to stimulate cells in a separate culture and perform a comparative secretome analysis (58, 59). The disadvantage of such an approach is that it only allows the study of unidirectional cell communication that does not take into account the reciprocal interactions that are likely to occur *in vivo*. Yet, the relative simplicity of such experiments can be creatively used to decipher cause and effect, as well as reciprocal effects, by taking the two-way signaling apart in single analyses.

To mimic a scenario of biological crosstalk, cells can be physically cocultured in the same well (60) or using a semipermeable trans-well system (61) to investigate cellular communication that requires cell-cell contacts or is mediated by soluble factors, respectively. This has been successfully used to elucidate factors secreted by one cell type upon coculture with another, which has been particularly informative when combined with an assay to monitor altered function of the recipient cell. For instance, in the seminal work investigating the ability of 23 stromal cell types to influence the resistance of 45 cancer cell lines to 35 anticancer drugs, it was revealed that induction of stromal-mediated drug resistance is a common phenomenon (60). It was found that one of the trans-activities could be attributed to HGF, which was identified in the conditioned media of fibroblasts using antibody arrays and next confirmed to rescue melanoma cells from the mutant-BRAF inhibitor PLX4720. The demonstration that combined treatment with inhibitors of BRAF and MET (the HGF receptor) can synergize in killing cancer cells (60) shows the potential of secretome analysis of coculture systems to identify factors that may rationalize novel treatment strategies. This was similarly achieved in a study revealing that cancer-associated fibroblasts confer platinum resistance to ovarian cancer cells, which was abolished by T-cell–derived IFNγ, leading the authors to suggest that immunotherapy may subvert chemoresistance and improve chemotherapy efficacy (61). Another study used cytokine antibody arrays to systematically investigate reciprocal cellular interactions by pairwise coculturing of prostate epithelial cells, prostate cancer cells, and fibroblasts, identifying multiple secreted factors that were altered in each case (62). Among these identified factors, follistatin secretion was induced upon coculture of epithelial cells and cancer cells, and follistatin promoted cancer cell motility, illustrating the complex function of the microenvironment-dependent secretome.

### Cell Type–Specific Labeling

Since in a coculture experiment it is not possible to determine from which of the two cell types a particular protein originates, several cell type–specific labeling strategies have been developed that allow this distinction to be made. One approach, called cell type–specific amino acid precursor labeling (CTAP), exploits the fact that L-lysine is an essential amino acid for mammalian cells. In particular, transgenic expression of plant and bacterial genes in the lysine biosynthetic pathway overcomes this auxotrophy by allowing conversion of lysine precursors into L-lysine (63). The power of the approach lies in the fact that different enzyme-precursor pairs can be engineered, so that each cell type in the coculture can metabolize only one of the lysine precursors and not the other. When supplying precursors that carry different stable isotope labels, the cell of origin can be deduced from the label status of the secreted (or intracellular) proteins as determined by LC-MS (63).

The CTAP procedure was further optimized by selection and sequence optimization of precursor-converting enzymes from different bacterial species (64). Besides achieving enhanced catalytic activity, a key merit was the strict intracellular retention of the enzymes, thus avoiding premature precursor conversion (64) that severely compromised coculture labeling efficiency before (63). This is an important advancement especially for secretome analyses, since it allows uninterrupted, continuous coculturing for extended periods of time (days) without requiring media exchange. CTAP has been used to characterize reciprocal signaling between fibroblasts cocultured with prostate cancer cells (65) or KRAS(G12D)-mutated pancreatic ductal adenocarcinoma cells (66), identifying multiple coculture-induced (phospho)proteomic differences in either cell type. In the latter study, the trans-acting effect was attributed to SHH secreted by pancreatic ductal adenocarcinoma cells, which had previously been identified as a candidate in the supernatant of these cells grown as a monoculture (66). Collectively, these studies have shown that CTAP can reveal the complex interplay between cells, in particular to uncover non-autonomous cellular processes at a level that was previously unrecognized. To deepen this understanding, direct analysis of secretomes obtained from such experiments is a still largely untapped opportunity to identify the factors that mediate this reciprocity, while providing candidates whose neutralization could abolish this effect.

An alternative approach to achieve cell-selective labeling is to express a mutant tRNA synthetase to incorporate non-natural (and most notably clickable) amino acids into nascent proteins. The principle entails the mutation of a specific aminoacyl-tRNA synthetase to enable its charging with a non-natural instead of the canonical amino acid. Although in principle this can be applied to various amino acids (67), variants of methionyl-tRNA synthetases (MetRS) have been most extensively explored for proteomic applications (68). To label mammalian cells, best results have been achieved by expression of the L274G-mutated mouse MetRS, allowing robust incorporation of azidonorleucine (ANL) into proteomes of mammalian cell lines (69). The azide moiety of ANL can then be used for click-based conjugation to alkyne-containing probes for visualization or enrichment of newly synthesized proteins. Importantly, this enables selective proteomic characterization of L274G-MetRS–transduced cells in coculture with nontransduced cells, where only the former will incorporate ANL. This extends to secretome analysis to identify ANL-tagged proteins in a cell-selective and coculture-dependent manner, even in culture media that contains serum (70). Taking this a step further, the system has been established in a mouse model to specifically label hippocampal neurons with ANL for subsequent proteome comparison of excitatory and inhibitory neurons or of neurons in mice in different sensory environments (70). It is readily conceivable that this can be extended to other cell types by conditional expression of L274G-MetRS in mice on an ANL diet. Using a complementary strategy, broad expression of L274G-MetRS resulted in the incorporation of ANL in multiple tissues, and, interestingly, tagged proteins could also be detected in serum, including many classical secretory proteins such as inflammation regulators and tissue remodelers (71). This opens the exciting possibility that cell type–specific proteins can be selectively enriched and identified from the blood circulation for biomarker discovery or, ultimately, for diagnostic purposes.

The recent demonstration that cell-selective ANL labeling can also be performed in orthotopic cancer models in mice further broadens this perspective to investigate protein synthesis and secretion in cancer cells in the context of their microenvironment *in vivo* (72, 73). This may be applicable to many cell line–derived xenograft models that have been developed in the past or even to patient-derived xenografts if the primary cells are amenable to lentiviral infection. Alternatively, ANL labeling in mouse cancer cells has the advantage that grafting of cells can be performed in immunocompetent mice, thereby encapsulating interactions with infiltrating immune cells that may affect the tumor secretome and ECM composition (73).

## Proximity Biotin Labeling in the Secretory Pathway

In recent years, proximity-dependent biotinylation has become an increasingly powerful approach to tag proteins that reside in a subcellular compartment of interest to determine its proteome composition. The concept relies on the fusion of a promiscuous biotin ligase to a compartment-specific protein to biotinylate proteins in its immediate vicinity, which can then be isolated by streptavidin-capture for subsequent identification by mass spectrometry (74, 75, 76). This has been tailored in recent studies for the characterization of secreted proteins both *in vitro* and *in vivo*. In particular, they use similar but complementary strategies by expressing the fused biotin ligase in the cell’s secretory pathway to biotinylate nascent proteins on their way to the extracellular space. For instance, Kim *et al* exploited the ER-resident protein Sec61 for biotinylation of secretory proteins during transit through the ER lumen, in liver cells both in cell culture and in mice (77). Interestingly, several dozen biotinylated proteins could be detected in plasma of these mice, of which bovine serum albumin was the most prominent, indicating that they originate from the liver. They then used their approach to identify circulating proteins in plasma upon induction of insulin resistance and identified 20 proteins that were absent in control mice, several of which had previously been associated with insulin resistance (77). Three other studies that appeared around the same time used the KDEL signal peptide to target biotin ligase variants to the ER lumen (78, 79, 80). Liu *et al* used KDEL-tagged BioID to target it to the ER and expressed it in mouse skeletal muscle to identify exercise-induced secretory proteins. They observed an increase in the abundance of the known muscle protein myostatin in plasma of mice after wheel running (78). Extending this to other cell types, Wei *et al*. used KDEL-tagged TurboID instead of BioID and expressed it in hepatocytes, myocytes, pericytes, and myeloid cells and characterized their secretome composition both in cell culture and *in vivo* by their enrichment from plasma in mice (80). These secretome profiles not only allowed them to distinguish cell types but also to identify the cell type of origin of some classic plasma proteins. Beyond these secretome catalogs, this study investigated dynamic alterations in the *in vivo* secretome, benefitting from the fact that biotinylation only commences upon administration of biotin. By analyzing plasma-derived liver secretomes in mice that received a diet of high glucose and fructose, they observed global suppression of protein secretion, accompanied by a strong increase in the secretion of a single protein, betaine–homocysteine S-methyltransferase, indicating a previously undescribed nutrient-dependent reprogramming of the hepatocyte secretome. An additional interesting aspect is that secretion of betaine–homocysteine S-methyltransferase was believed to occur *via* unconventional export, since it was detected using a strategy that targets TurboID to the cytoplasmic side of the ER (80). Yang *et al.* used an engineered promiscuous biotin ligase (BirA∗G3) and showed that it could identify proteins known to be secreted by both conventional and unconventional mechanisms (81). They targeted it to various body parts in *Drosophila* (brain, fat body, muscle), in each case identifying organ-specific factors including several with a hormonal or signaling function (79). They also applied this strategy in mice to express the biotin ligase in teratomas, showing that several of the teratoma-derived secretory proteins were also found in serum (79).

The rapid succession of the above publications demonstrates the necessity and timeliness of investigating the nature and function of secreted proteins *in vivo*. Although conceptually similar, these studies show great versatility in the choice of biotin ligase (BioID, TurboID, BirA∗G3), the tissues and cell types that can be targeted, and the physiological and disease processes that can be studied. A potential challenge in these analyses is the formal confirmation of the biotinylation status of secreted proteins identified in plasma, thus suggesting their tissue origin, which can only be obtained from direct mass spectrometric identification of a biotinylated peptide. This may not be straightforward since recovery of these peptides from streptavidin beads can be low, but on the other hand, this may be less relevant in comparative studies as long as quantitative secretome differences can be observed between the conditions that are studied. Therefore, the concept of proximity-dependent biotin labeling should be very useful in many biological scenarios to understand the function of secretory factors in inter-organ communication (82). This may not always need sophisticated tagging approaches, as shown by the identification of novel myokines and adipokines identified in the extracellular fluid from muscle and fat tissues of mice (83).

## Exploiting Protein Glycosylation to Characterize the Secretome

### Glycan-Directed Secretome Analysis in Serum-free Conditions

Proteins that are secreted through the classical secretory pathway are usually glycosylated during protein maturation in the ER, and this property can be exploited for the isolation and characterization of proteins in the secretome. Classically, enrichment of glycoproteins and peptides has been performed with hydrazide chemistry (84), different flavors of lectins (85, 86), immobilized metal affinity chromatography, or by hydrophilic interaction chromatography (87, 88). These concepts have been extended to the analysis of secretomes and cell surface proteins, both *in vitro* and in plasma in animals (89), as reviewed elsewhere (90, 91). For instance, lectin-based enrichment has been combined with SILAC labeling to compare proteome profiles of conditioned media obtained from different breast cancer cell lines, where secretome composition could be associated to the cancer stage represented by the used cell lines (92). Interestingly, several of these proteins could also be detected in plasma, showing the potential to identify tissue-derived proteins in the circulation, which may have clinical or diagnostic relevance (92). Another study used hydrophilic interaction chromatography and label-free proteomics to compare secretomes of four human hepatocellular carcinoma cell lines with different metastatic potential. They identified differential secretion of multiple proteins with a function in cell motility and migration but also in various other cellular processes (93). Several studies performed glycan-directed secretome analyses to identify substrates of proteases and proprotein convertases that are shed into the extracellular space to mediate cellular behavior, especially in cancer. For instance, Duval *et al* identified shedding of CASC4 in the conditioned media of cells expressing PC7/PCSK7, establishing it as a novel substrate of this endopeptidase. In addition, they showed that shedding of CASC4 resulted in enhanced cellular migration and invasion, thereby explaining (at least in part) the protumorigenic role associated with PC7 (94). Both studies highlight the potential of secretome analyses to identify regulatory processes in cancer metastasis.

### Glycan-Directed Secretome Analysis in Serum-Containing Conditions

Although all of the above studies successfully profiled cellular secretomes using glycans as a handle, they have the shortcoming of requiring serum-free cell culture conditions, potentially affecting cellular properties or even survival. To circumvent this, strategies have been designed to introduce clickable reactive groups in glycan moieties for selective isolation of cell-derived secretory proteins from serum-containing cell culture media (88). In particular, externally supplied azido-sugar analogs of sialic acid (tetraacetylated N-azidoacetyl-d-mannosamine; ManNAcAz) or N-acetylgalactosamine (tetraacetylated N-azidoacetyl-d-N-acetylgalactosamine; GalNAcAz) can be metabolically converted and incorporated into N- and O-linked glycans (95). This was used by Lichtenthaler *et al* in a method termed SPECS (‘secretome protein enrichment with click sugars’) to enrich cell-derived proteins from culture supernatants by reacting them with alkyne-biotin (96). With subsequent streptavidin pull-down and LCMS analysis, they identified several dozen membrane proteins whose shedding in the extracellular space depended on the activity of BACE1 (beta-secretase), a proteolytic enzyme mediating the liberation of amyloid precursor protein from neurons, and a key drug target for Alzheimer’s disease. In another study, these authors used SPECs to determine that substrates of signal peptide peptidase-like 3 (Sppl3) spanned various proteins involved in N- and O-glycosylation in the secretory pathway, thereby implicating a general role in Golgi function (97). These studies indicate how substrates of secreted proteases can be characterized to elucidate their function but also to understand potential side effects of such drugs that target these enzymes.

More recently, the same group implemented an improved ‘high performance’ version of this method (hiSPECS), by including a lectin-based glycoprotein enrichment step, direct coupling of azide glycoproteins to alkyne beads thus omitting biotin-tagging, and on-bead proteolysis (98). This allowed them to improve overall sensitivity and to downscale the experiment by 40-fold, identifying novel BACE1 substrates not found in their earlier study (96). In addition, they determined cell type–specific secretome profiles of primary astrocytes, microglia, neurons, and oligodendrocytes, covering close to 1000 proteins that included both soluble secreted proteins and ectodomains of shed membrane proteins, many of which could also be detected in cerebrospinal fluid, indicating that they could serve as diagnostic markers of disease or even of cell-of-origin. A similar approach has been used to determine secretome alterations after activation of T-cells (99).

### Glycan-Directed Secretome Analysis with Isotope Labeling

The studies discussed above used label-free approaches for protein quantification; however, the use of stable isotope labeling provides distinct advantages in terms of accuracy and reproducibility, although this has not been used frequently in combination with glycan-directed secretome analysis. Xiao *et al.* showed that SILAC labeling can be integrated into a workflow for the differential quantification of glycosylated cell surface proteins (100), hence this should also be applicable to secretome analysis. TMT labeling has been used to boost the number of detected secreted proteins by running a higher-input secretome sample within the TMT series (101), thereby following a similar strategy as used in single-cell proteomics (102). Employing TMT labeling and click chemistry–based enrichment of glycosylated proteins, a boosting-to-sample ratio of 10:1, tripled the number of identified proteins in the secretome obtained from cells that were cultured in the presence of serum (101). This number was still lower to the coverage obtained from serum-free media, which was attributed to the difference in sensitivity between the ion trap and the Orbitrap used for the detection of the TMT reporter ions. Yet this allowed the authors to identify LPS- and TGFβ-induced secretion of various proteins, including cytokines, growth factors, and ECM proteins (101). Since SILAC labeling has been effectively used in combination with metabolic labeling with clickable amino acids, a deeper exploration of similar strategies employing clickable sugars may be advantageous to expand the scope of secretome coverage or, when used in combination, to investigate the role of glycosylation of secretory potential of distinct targets.

### Critical Note on Glycan-Directed Approaches

These studies have shown that glycans can be effectively targeted to define the secretome, albeit still with limitations. Firstly, coverage of the glycan-modified secretome depends on the choice of lectin. Given the different glycan specificities of each lectin, the relative proportion of glycan-modified proteins retrieved will differ and can be misleading if interpreted on its own. To overcome this, lectin mixtures may offer the best approach. Along the same lines, when relying on click sugars, only proteins that have sialic acid or GalNAc modifications will be labeled. It should be critically noted that such strategies are also unlikely to be exhaustive in retrieval. More importantly, secreted proteins enriched *via* glycan-directed approaches cannot be compared in relative abundance to the unmodified secreted protein if such forms co-exist in the biological secretion, due to the enrichment bias.

## Post-translational Modifications of Secretory Proteins

In traversing the ER and Golgi apparatus, secretory proteins undergo a variety of posttranslational modifications, ranging from disulfide bonds necessary for protein structure to many forms of N- or O-linked glycosylation and also phosphorylation, hydroxylation, or acylation, among others (103). These modifications not only ensure faithful progression through the secretory system but often also determine maturation of secretory cargo towards bioactive molecules (104). In addition, once secreted in the extracellular space, proteins can undergo further processing by proteases and other enzymes that have been secreted themselves and that alter or neutralize their activity (39, 105, 106, 107). While recent proteomic studies have begun to elucidate the extent and diversity of post-translational modifications (PTMs) on secretory proteins, understanding the mechanisms by which this occurs and correlating this to the biological function and activity of their substrates is a task that still awaits to be fulfilled for the majority of proteins observed in the secretome.

### Glycosylation

Apart from using protein glycosylation as an affinity handle to enrich for secretory proteins (see above), it is important to note that this modification itself is a crucial regulator of protein function and activity. Therefore, an important task of secretome analysis is to determine the site and nature of glycosylation events during and after maturation of secreted proteins. Glycosylation is one of the most diverse posttranslational modifications that functions in folding, quality control, stability, transport, and activity of a wide variety of substrate proteins. Glycosylation mainly occurs in the ER and the Golgi system involving around 200 glycosyltransferase enzymes that act in a concerted manner, resulting in the glycosylation of most (>85%) secretory proteins (108). This furnishes a machinery for tightly controlled and tailored protein glycosylation in cell condition–dependent manner, which often has been studied for individual proteins due to mechanistic complexity. Well-known examples are the site-specific O-glycosylation of fibroblast growth factor 23 (FGF23), granting its secretion (93), and the multiple types of O-glycosylation that are required for the function of the NOTCH receptor (109). Proteomic studies have been conducted to globally catalog the repertoire of glycosylated proteins in the secretome in various cell systems, typically employing lectin-based enrichment of glyco-peptides obtained after proteolytic digestion, sometimes combined with neuraminidase or PNGase F treatment to cleave sialic acid or N-glycans, respectively, to simplify subsequent mass spectrometry while allowing site-localization of glycans (110). This has allowed the identification of N- and O-glycosylation sites in hundreds of secretory proteins, providing great insight in the functional diversity of proteins undergoing this modification and in the domain preference where this occurred (93, 109, 111, 112). Yet, the identity and branching structure as well as functionality of these modifications typically remain elusive due to the global nature of these profiling experiments.

Taking this a step further, a recent study (113) combined proteomic and functional approaches to first discover and then delineate how enhanced fucosylation of secreted proteins underlies resistance to osimertinib, a third-generation EGFR-tyrosine kinase inhibitor. Among the proteins exhibiting tyrosine kinase inhibitor, treatment-induced fucosylation was the antioxidant protein PON1, prompting its secretion and stabilization. In turn, this induced a gene expression program of transcription factors and gene effectors that could be associated with drug resistance (113). This is just one example illustrating the intricate mechanisms of how glycosylation events in secretory proteins mediate drug response, at the same time providing potential novel inroads to intervene in this process and curb the highly relevant clinical process of therapy-induced drug resistance.

Despite recent advances in mass spectrometry–based glyco-analytics, the sensitivity bottleneck remains for a full documentation of diverse glycoforms in the secreted protein repertoire. Indeed, protein capture with single lectin species may also introduce a bias and preclude a true reflection of the stoichiometry of a glycan-modified secreted protein. In this respect, the combined use of multiple lectins for glyco-peptide level enrichment (114) and diagnostic mass triggering MS acquisition (115, 116) may overall achieve better sensitivity and specificity of detection. In an ideal scenario, glycosylation events may evolve as biomarkers in the development of novel disease stratification tools (117).

### Phosphorylation

Although more than 500 kinases are encoded in the human genome to regulate a plethora of cellular processes, only two have been found localized in the secretory pathway (118), one of which is a protein kinase (vertebrate lonesome kinase) that phosphorylates proteins traversing the secretory pathway and proteins in the extracellular space (119). Yet, the majority of phosphorylated proteins in the secretome contain a phospho-motif of SxE/pS, which was found to be mediated by a single kinase, FAM20C (120). FAM20C is a casein kinase expressed in the Golgi apparatus estimated to phosphorylate more than 100 genuine secretory substrates with diverse functionalities (120). Interestingly, phospho-proteome analysis of blood plasma indicated that 58% of the high-confidence phospho-sites contained the SxE/pS motif (121), including sites that modulate the activity of factors acting in blood coagulation (120, 122). In addition, different secretory protein substrates were found to be phosphorylated depending on the cell type, for example, in beta-cells, cardiomyocytes, and bone cells (118). This all indicates that FAM20C has a wide-spread and diverse role in modulating the signaling activity of secreted proteins.

### Proteolytic Processing

Proteases mediate the degradation of proteins; however, their activity across a range of specialized enzyme classes are increasingly appreciated to precisely regulate protein processing that can modulate signaling activity of crucial proteins (123). This includes several extracellular proteases with diverse substrates such as growth factors, receptors, and matrix proteins to modulate cancer progression, inflammation, or other diseases (124, 125). To understand protease specificity and gain insight in protease-substrate relationships, tailored mass spectrometry–based methods have been designed to determine neo-N-termini that arise upon protease treatment at a proteome-wide scale (126). Among these, terminal amine isotopic labeling of substrates is a popular method that blocks primary amines (*i.e.* N-termini and Lysine side chains) upon incubation with the protease of interest, followed by tryptic digestion. At this stage, peptides containing neo-N-termini should be blocked, which can be negatively selected by capturing primary amine–containing peptides through a reductive amination reaction with a hyperbranched polyglycerol-aldehyde polymer (127). Thousands of neo-termini can be identified in a single experiment, which can be done in a quantitative manner when performing the blocking step with a heavy isotope-containing reagent (*e.g.* TMT or iTRAQ) (127). This approach has been used to profile proteins undergoing proteolytic cleavage in a tissue context and, more relevant for secretome characterization, has been performed to determine target landscapes of individual secreted proteases, including the metalloproteinases MMP2 and MMP9 (128), ADAMTS2, ADAMTS3, and ADAMTS14 (129), ADAMTS7, ADAM10, and ADAM17 (130). Interestingly, for ADAMTS2 and ADAMTS14, this has also been performed *in vivo* in the skin of WT mice and animals that were deficient of these enzymes (131).

In a cancer context, terminal amine isotopic labeling of substrates was used to understand the impact of malignant transformation on protein processing in the secretome, observing the processing of growth factors and the ECM, with potential implications on signaling events regulated by protease substrates (132). In a global secretome analysis to understand the function of the secreted protease PRSS35, the study found that PRSS35 inhibits cancer progression through cleavage of the chemokine CXCL2, thereby interfering with protumor neutrophil function. Interestingly, and as a cautionary note, this effect could only be observed *in vivo* because of the involvement of both cancer cells and neutrophils and not in mono-culture of cancer cells *in vitro* (132). Finally, mass spectrometric analysis of intact proteins (top-down proteomics) is emerging as a mature technology that is ideally placed to identify distinct proteoforms, that is, proteins that have undergone distinct PTM and processing events. Applied to secretome analysis of cells expressing HRAS-G12V to induce a senescence-associated secretory phenotype (133), this identified 55 proteins and 258 proteoforms, including several new proteoforms of known senescence-associate secretory phenotype factors (IL8, CXCL5, CXCL2, HMGA1, and S100A13). Specifically, the authors found five distinct IL8 proteoforms. These contained cysteine S-sulfhydration or S-sulfinic acid events, PTMs that had not been previously identified on IL8 (133). This illustrates the complexity of secretome composition that was not appreciated before, the functional implications of which remain to be explored.

In a human organismal setting, the enteroendocrine cells in the human gut are responsible for hormone production, secretion, and protease-dependent processing. Some of these hormones can regulate glycemia, appetite, and satiety (134), for instance ghrelin, cholecystokinin, glucagon-like peptide 1, peptide YY, insulin-like peptide 5, and oxyntomodulin. Two recent consecutive studies have generated the first human enteroendocrine cell hormonal atlas (135) by analyzing the cleaved pro-hormone products and ascertained the protease specificities required for fundamental processing of pro-hormones into functional peptide hormones (136). Collectively, these demonstrate a niche application of mass spectrometry–based secretomics in characterizing hormonal peptide processing that cannot be achieved by RNA sequencing.

### Multiple PTMs in the Extracellular Matrix

A cellular structure where many of the above PTMs come together is the ECM, the acellular component that provides mechanical support to all tissues and organs which also fulfills a crucial function in development, tissue repair, and cell migration (10). The ECM consists of an amalgam of collagenous and noncollagenous proteins, glycoproteins, hyaluronan, and proteoglycans that assemble into insoluble entities. Collectively, this is also referred to as the matrisome, which may represent the most ubiquitous group of secreted proteins estimated to account for ∼4% of the human proteome (137) and comprising approximately 20% of the adult brain volume (138). The matrisome is abundantly glycosylated but is decorated with multiple other PTMs (139). For instance, proteomic analysis of ECM-enriched material from pancreatic cells identified 214 matrisomal proteins, where N-glycosylation was detected on 99 proteins, phosphorylation on 18 proteins, and nine proteins carried both PTMs (140). In a study of mouse mammary tumors, 225 unique ECM proteins were identified carrying a total of 229 PTMs that included glycosylation, phosphorylation, and hydroxylation (141). In fact it is the latter modification that plays an important role in giving the ECM its rigidity, where hydroxylation of proline and lysine residues is carried out by a range of ER-resident enzymes vitally contributing to the maturation and assembly of fibrillar collagen molecules (139). In addition, lysyl oxidases mediate collagen cross-linking, resulting in stiffening of the ECM, which underlies pathological processes in cancer development by promoting tumor cell invasion and metastasis (105, 142). Stiffened ECM also present as physical barriers to the access of therapeutic agents to the tumor cells (143). Collagens undergo extensive proteolytic cleavages during conversion of pro-proteins to allow subsequent fibril formation and the shedding of ectodomains in the extracellular space to modulate cell adhesion (137). In addition, the ECM serves as a reservoir for multiple growth factors and cytokines that can be liberated upon ECM degradation, adding an additional layer regulating the bio-availability of these factors to drive disease or maintain tissue homeostasis (137). Given the key role of the ECM in health and disease, excellent reviews are available describing current insights in ECM biology and its potential for therapeutic targeting (10, 144, 145). Similarly, proteomic approaches to characterize ECM composition has been covered in various recent reviews, describing sample preparation and mass spectrometric methods that address the challenge of mechanical resilience and molecular heterogeneity of the ECM (6, 138, 146, 147). To truly reflect and understand the modification diversity in the ECM in health and disease, a multi-PTM analytical approach is critical.

## Hypothesis-Driven Secretome Analysis

Although mass spectrometry is the method of choice for unbiased secretome analysis, other approaches may be advantageous for hypothesis-driven secretome profiling. For instance, antibody-based, bead-based, membrane-based, DNA barcoded, or linker-based formats have been designed for the focused assessment of cytokines and growth factors. Examples of these are cytokine arrays and antibody panels that can be assembled based on a pre-definition of secreted analytes of interest. Numerous commercial platforms are available, and in recent years, even hybrid protein, cytokine, and RNA detection has been achieved in readouts from single wells. Using such strategies, biological investigations have revealed the impact of secretions on human mesenchymal stem cells in models of Parkinson’s Disease (148), mechanisms of tissue regeneration (149), and stromal effects on joint health (150). The clear advantages of such assay formats are detection specificity and customizability. Yet, the cost of doing so remains relatively high with limited to no capabilities for multiplexing beyond 2 to 3 samples. Truly novel secretion would also be excluded *a priori* by the panel design as a necessary trade-off for specific monitoring of a desired target list. It is also important to note that the linear range of such arrays are typically determined by the linear range of antibody or interaction-based detection and would be much smaller than quantitative mass spectrometry readouts.

## Secretion and Communication *via* Extracellular Vesicles

Secretion of proteins *via* EVs provides a major route for cellular interaction, operating in parallel to classical and non-classical secretion mechanisms. The biogenesis of EVs is complex and can broadly be categorized by the ultimate size of vesicles that will be produced (151, 152). Small EVs (exosomes) are generated by inward membrane invagination, endosomal fusion, and then maturation into multivesicular bodies which fuse with the plasma membrane to release their cargo in the extracellular space. Larger EVs and microparticles are generated instead by outward membrane budding that shed mostly cytoplasmic material. These key differences in biogenesis path can therefore impact the logical protein cargo exported by different kinds of EVs. Endocytosis can introduce extracellular contents into exosomes, and the acidic environment generated after endosomal fusion can shape the cargo content by pH stability. This, for instance, has an impact on the antigen peptide binding on HLA class I molecules inserted in the EV membrane (153). All in all, these key differences in biogenesis make the protein cargo from EVs (both exosomes and microparticles) very diverse and poorly predictable by classical means. At the same time, there is increasing awareness of the important role of EVs in cell communication, and therefore this is a very active field of research, both in basic and clinical biology. Instead of attempting to cover this emerging field, we refer to excellent recent reviews that cover aspects both on the function of EVs in tissue homeostasis and disease (152, 154, 155, 156) and on methods for their isolation and proteomic characterization (157, 158).

The one particular aspect of protein secretion by EVs that we like to highlight here is that protein cargo is stabilized by protecting it from proteases and other enzymes in the extracellular space, thereby clearly distinguishing this from secretion of free protein. Specifically, the EV membrane can encapsulate the intravesicular protein (and lipid and RNA) contents in a relatively controlled environment to survive long distance transit for distant signaling and crosstalk and deliver functional protein cargo to recipient cells. For instance, exosomal secretion from single cells is now quantifiable using a pH-sensitive GFP (pHluorin) reporter system and shown to directly modulate GPCR signaling (159). A M153R mutant version of the same live-cell reporter has been used to investigate the role for exosomes in promoting leader-follower behavior in 2D and 3D migration (160). These are excellent tools to understand the secretion dynamics, functional impact, and paracrine roles of EVs in promoting cell migration and cancer metastasis. Lipid membrane encapsulation of EVs also allows seamless integration of the resting or activated cargo protein receptors into the plasma membrane of the recipient cell in the right membrane topology. When packaged and released in exosomes, ligand receptors are protected during transit through the extracellular space until uptake in recipient cells. There, with membrane fusion, receptors in the EVs are returned to the correct topology and indistinguishable from resident receptors originally present in the recipient cell. For instance, by investigating the phosphoproteomes from isolated EVs, it was shown that triple negative and HER2-positive breast cancer cells produce EVs that can recapitulate the respective phosphorylation signaling in each disease subtype (161). These allude to the functional impact of receiving phosphorylated receptors, in priming for distant metastatic spread.

## Computational Methods and Resources for Secretome Analysis

While mass spectrometry is largely a discovery science, the secretome community has adopted stringent quality controls for mass spectrometry data. The gold standard in secretome profiling is to report the percentage of secreted proteins among total proteins identified, in addition to detection of known secreted protein in an experimental system. For instance, the latter could be gastrin from cells of the stomach or insulin from endocrine cells in the gut.

The relatively conserved nature of signal peptides allows for computational prediction of protein secretion, for instance, with SignalP (162) and SPdb (163), using the primary polypeptide sequence as input. In the recent years, deep learning has been further applied on existing knowledge, to improve the computational annotation of signal peptides (164). Protein language models have also been applied to recent prediction database upgrades, enabling the detection of all five signal peptide types from metagenomics data (165). The caveat in doing so raises biological implications that although a protein may have inherent sequence features that can support secretion, a cell is still selective in time and space with its actual secreted repertoire. Clear examples of these would be in the production of neurotransmitters (166, 167, 168), during lactation (169, 170) and hormone secretion and processing in the gut (171). A Signal Peptide Secretion Efficiency Database (172) has also been assembled to guide the choice of signal peptide in engineering products of secretion to prioritize effective extracellular product accumulation (173, 174).

Classical secretion typically makes up about 60 to 70% of the detectable secretome. The remaining may be rationalized by other mechanisms including receptor ectodomain shedding and EV secretion. SecretomeP annotates non-classical secretion, by augmenting the predictions with posttranslational and localization aspects of the protein (175). Since non-classical secretion mechanisms diverge between human and microbes, the prediction guiding principles are also different. Along the lines of such non-classical prediction, secreted proteins also tend to feature more glycosylations and disulfide bridges for added stability (176). As such, although not fully predictive, the presence of such modifications and modification sites may also indicate higher confidence in *bona fide* protein secretion.

Unlike the sequence-driven nature of classical secretion, packaging and exporting a protein *via* EVs or exosomes appears to be much less predictable and highly dependent on the cellular state (177, 178) and physiological stimuli (179). Therefore, documentation of the extracellular vesicular protein cargo has proceeded largely *via* compendiums from actual mass spectrometry and experimental observations. For this purpose, Exocarta (180) and Vesiclepedia (181) have become the central point of data deposition. By virtue of a prior observation in EVs, one gains confidence that the detection of the same protein in another isolate of exosomes is coherent. Considering that EV production and contents can change with different cellular states, extrapolating possible exosomal secretion requires caution, not to mention the stringency of vesicle preparation before acquiring these deposited datasets can also influence experimental annotation. The quality standards of EV and exosome analyses are collectively defined by the Minimal Information for Studies of Extracellular Vesicles (MISEV) guidelines (182), which was extensively reviewed elsewhere (183). As the sensitivity of mass spectrometers and cumulative number of secretome reports increases, the proportion of non-classical secreted products also appears to increase, often inviting the question if this is a real gain in knowledge of protein export or an artefactual gain in cytoplasmic contaminants being picked up by ultra-sensitive instruments. Hence, benchmarking the proportion of secreted protein in a secretome dataset requires a sensible approach that balances a realistic estimate of the secreted protein subset, the secretory phenotype of the model system, and the experimental scale against sensitivity depth.

In general, a secretome dataset becomes more trustable, with higher proportion of annotated secreted proteins, based on *in silico* and curated database information. Using a combination of annotation sources, a good secretome preparation should exceed 90% in composition with secreted proteins. This sample purity will be heavily impacted by loss of cell membrane integrity during culture and preparation and the resulting contamination with cytoplasmic proteins. It is important to note that such mixing of intracellular and extracellular proteins cannot be overcome by any form of metabolic labeling strategy, as tags like SILAC or AHA will inevitably label intracellular proteins first before secretion.

## Challenges and Future of Secretome Analysis

Despite the astounding progress that has been made in characterizing secretomes, several technical hurdles and biological considerations remain. BONCAT has been such a successful technique for secretome labeling, yet some cell types (for instance neurons) are known to be very sensitive to methionine starvation and cannot tolerate even 30 min of methionine deprivation in culture. In metabolically active cells, this is about the minimum duration needed to deplete the intracellular methionine depot (>90%) with resting new protein synthesis, as verified by LC-MS–based metabolomics (59). In such cases, direct supplementation of AHA at 5 to 10 times the physiological methionine concentration can somewhat skew the biosynthetic incorporation towards AHA without the methionine starvation step (184). Other instances of AHA toxicity have also been reported, where prolonged exposure to AHA may induce proteome alterations (185). In these cases, secretome analyses may be more suitable to proceed *via* other approaches. Another limitation that also stems from methionine replacement is the intracellular recycling of methionine. Methionine is an essential amino acid initiator that needs to be imported *via* neutral amino acid transporters, and it is required to kick start translation. Free methionine amino acids regenerated from turnover of existing proteins (before pulsed AHA labeling) may systematically re-enter the intracellular biosynthetic pool and become re-incorporated into nascent proteins. This results in reduced AHA labeling efficiency, which may be misinterpreted as poor click chemistry efficiency, since the procedure of on-beads digestion makes the experimenter blind to the actual AHA incorporation rate. It is particularly important to consider the impact of such recycling when designing longer AHA labeling experiments. Yet, labeling of nascent proteins with clickable methionine analogs remains a powerful concept especially if it aims to determine differences in secretion profiles between cellular conditions that will be validated in subsequent experiments. In this respect, it will be worth exploring the performance of clickable variants of other amino acids, such as β-ethynylserine, a bioorthogonal analog of threonine that has recently been shown to be efficiently incorporated in nascent proteins (186).

In many cases of purifying AHA-labeled secreted proteins, biotin-tagged alkynes are the preferred approach, as streptavidin can retrieve the clicked proteins very efficiently and specifically. The caveat is however the strong affinity between biotin and streptavidin, which has a low efficiency of release even with strong harsh eluting conditions. Direct digestion of captured proteins directly off streptavidin beads is not a viable option because of the massive release of streptavidin-derived peptides, which has been circumvented by chemical modification of streptavidin rendering it resistant to tryptic cleavage (187). Alternatively, elutable biotins have been designed. Desthiobiotin has the same specificity to streptavidin compared to biotin but weaker affinity, such that elution from streptavidin can still be achieved by competing biotin. SS-biotins on the other hand contain a reducible disulfide bond within the molecule to achieve analyte elution by simple reduction. Recently, an alternative approach called PhosID has also been introduced, where a phosphonate-alkyne handle replaces the biotin-alkyne to retrieve azide-labeled proteins (188). Phosphonate resembles the structure of a natural phosphate, except for P-C bonds, hence phosphonate-alkyne–clicked secreted proteins can be retrieved by standard phospho-enrichment strategies like iron or titanium dioxide–based capture. The beauty of this retrieval system is that biotinylation sites are retained, since the modified peptides are directly enriched and analyzed just like phosphopeptides and phosphosites. The specificity of phosphonate-alkyne retrieval is very high (>90%), and thousands of peptides and proteins can be identified in each experiment, with spectral evidence of biotinylation. The utility of phosphonate handles extends beyond BONCAT but also into site-specific activity–based protein profiling (188).

Glycosylation is prevalent in secreted proteins to maintain stability outside the cell. One current limitation lies in the quantification of secreted glycoproteins. Purification of glycoproteins is inefficient due to steric hindrance. Capturing glycan-modified peptides after digestion using one particular lectin may mean that information about other co-occurring posttranslational modifications on the same secreted proteins are lost. There may also be a spread of intensity and abundance between a range of glycoforms, each with differential affinity and bias to any glyco-enrichment method. The consequence is that any glycosylated secreted protein cannot be quantified relative to the pool of unglycosylated and differently glycosylated species of the same protein. Fundamentally, this precludes the mechanistic understanding if a change in glycosylation is related to processing or stability. This leads to the ultimate key point that in characterizing the secretome, one needs to go beyond merely cataloguing the modified and secreted proteins and move towards a biological understanding of the signaling function mediated by secreted factors. To achieve this, experimental models for expedited biological testing of paracrine signaling are urgently needed. Ideally, these will employ *in vivo* models and sophisticatedly engineered for cell type–specific tagging to probe a full-scale organismal level of secretion, targeting, and biological crosstalk (189).

## Conflict of interest

The authors declare no competing interests.
